# Knowing Each Random Error of Our Ways, but Hardly Correcting for It: An Instance of Optimal Performance

**DOI:** 10.1371/journal.pone.0078757

**Published:** 2013-10-30

**Authors:** Loes C. J. van Dam, Marc O. Ernst

**Affiliations:** 1 Department of Cognitive Neuroscience, Universität Bielefeld, Bielefeld, Germany; 2 Cognitive Interaction Technology Center of Excellence, Universität Bielefeld, Bielefeld, Germany; 3 Bernstein Center for Computational Neuroscience, Tübingen, Germany; University of California, Merced, United States of America

## Abstract

Random errors are omnipresent in sensorimotor tasks due to perceptual and motor noise. The question is, are humans aware of their random errors on an instance-by-instance basis? The appealing answer would be ‘no’ because it seems intuitive that humans would otherwise immediately correct for the errors online, thereby increasing sensorimotor precision. However, here we show the opposite. Participants pointed to visual targets with varying degree of feedback. After movement completion participants indicated whether they believed they landed left or right of target. Surprisingly, participants' left/right-discriminability was well above chance, even without visual feedback. Only when forced to correct for the error after movement completion did participants loose knowledge about the remaining error, indicating that random errors can only be accessed offline. When correcting, participants applied the optimal correction gain, a weighting factor between perceptual and motor noise, minimizing end-point variance. Together these results show that humans optimally combine direct information about sensorimotor noise in the system (the current random error), with indirect knowledge about the variance of the perceptual and motor noise distributions. Yet, they only appear to do so offline after movement completion, not while the movement is still in progress, suggesting that during movement proprioceptive information is less precise.

## Introduction

The human sensory and motor systems are less than perfect, due to noise inherent at every stage in the sensory and motor planning and execution pipeline [1]. For instance, when we try to point with our index finger to a previously seen target without visual control (i.e. open loop) our finger does not land exactly on target but there is a discrepancy between the pointing and target locations (pointing error). In order to improve or to optimally integrate signals from the different senses to make optimal decisions, knowledge about those errors is needed. Bayesian modeling approaches have shown that the only knowledge needed to perform optimally in most tasks, is an estimate of the overall distribution of the errors and in particular its variance [2]–[9]. However, this leaves unaddressed to what extent humans have knowledge of their own noise on a trial-by-trial basis, that is, for each individual movement. The very fact, that the noise reveals itself in the variable outcome means that at least no corrections for the noise occurred while performing the movement, even though corrections for the noise would lead to better pointing performance in terms of precision, which is what humans generally optimize in most sensorimotor tasks [10]–[14]. There are several different possibilities why humans do not correct for the sensorimotor noise on each movement. First, it is likely that humans have no knowledge of the noise in their system on a trial-by-trial basis and therefore, are incapable of correcting for it. Secondly, they could be aware of the noise and the resultant error, but do not have the time to correct for it online. It is generally known that the more time given to complete a movement, the more corrections can occur online and the better performance will be in both accuracy and precision (speed-accuracy tradeoff which in the framework of optimal control can be represented as the competition between two opposing cost-functions).

Here, we investigated the knowledge humans have of their own trial-by-trial random error caused by noise after each individual movement is complete without visual feedback. It is important to note that, in order to be as precise as possible, any information humans do have with respect to the noise or the random error on any ongoing movement, should be used to correct the ongoing movement online. Correcting the movement means reducing the error up to the point that information about any remaining error is lost. In other words, if we assume humans have access to their own noise instances, or at least the resulting error through sensory feedback, and there is time to use that knowledge online, we would predict people not to be able to report on their random errors at the end of each individual movement since this knowledge would already have been used to correct the movement online.

To this end, we investigated human pointing performance for both fast and slow movements, and both without and with varying degrees of visual feedback, and we asked participants after movement completion to indicate the direction of the error they had made (left or right of target). Different degrees of visual feedback were used in order to quantitatively determine the increasing influence on error knowledge. Surprisingly, participants were very able to give the correct response with respect to their own varying random error even without visual feedback. Moreover, we show that in spite of this knowledge, people do not correct online for the errors caused by the noise even when moving very slowly. We compared these results to an ideal observer model and demonstrate that this behavior actually reflects optimal behavior under the assumption that random errors can only be accessed with some precision at movement end-point.

## Results

**Figure 1:**
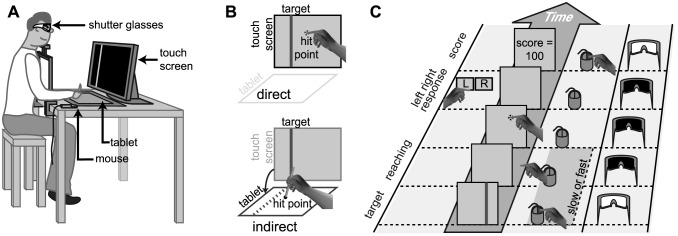
Experimental design and setup. A) Participants were seated in front of a touch screen on which targets were displayed. Participants performed pointing movement either towards the touch screen with their index finger or on a graphics tablet using a stylus. To control for visual feedback participants wore shutter glasses that were only transparent as long as a mouse button was being held. A chin rest restricted head movements. B) The complexity of the visuomotor mapping depends on the response mode (direct–touch screen vs. indirect–graphics tablet). Direct condition (top): for touch screen responses the pointing movement is directly towards the visual target location. Indirect condition (bottom): for tablet responses the pointing movement is on the horizontal plane of the tablet involving an additional mapping from the vertical image screen to the horizontal response plane. C) Sequence for a single trial. Participants initiated stimulus onset and shutter glasses transparency by pressing the right mouse button, such that with the onset of the movement visual feedback was prevented. After movement completion participants indicated whether they thought they had landed left or right of target. At the end of each trial they received a score based on absolute pointing accuracy.

Participants performed a 1-dimensional pointing task (see Figure 1). They were presented with a target that consisted of a vertical line on a touch screen. The touch screen was also used to record the pointing end-point positions. As soon as participants initiated the movement towards the target, further visual feedback of both the target and the arm movement was prevented using shutter glasses. After the movement was complete, participants were asked to indicate whether they thought they had landed to the left or right of target, either in the absence of visual feedback (Experiments 1 and 2) or with varying degree of visual feedback (Experiment 3). This judgement tested whether participants had knowledge about their random pointing error. In order to prevent participants from making deliberate errors rendering their left/right-judgements easier, they were motivated to point as accurately as possible to the target by rewarding them with a score after each trial. This score was based on their absolute pointing error (and thus did not provide directional feedback). On each trial, participants could score 100, 50 or 25 points depending on their error being less than 1, 2 or 3 cm respectively. If their error was bigger than 3 cm, they received no points. The final score for each experimental block was added to a high-score list to further motivate participants to point accurately.

**Figure 2:**
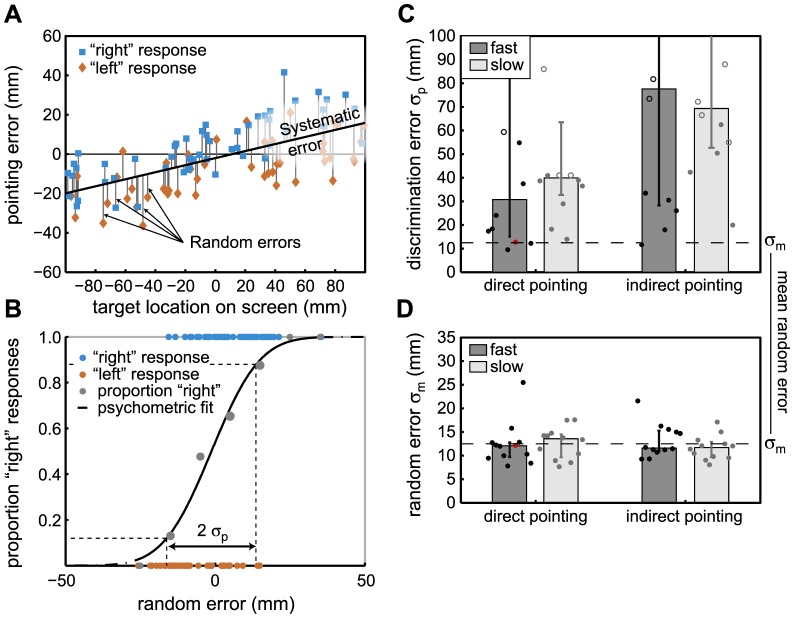
Results Experiment 1: fast and slow pointing in the direct and indirect pointing conditions. A) End-point error versus target location for an example participant in the fast pointing condition. The different symbols, indicating the response (left or right of target) for each trial, are clearly segregated with respect to the random error and not with respect to systematic errors. B) Psychometric curve (cumulative Gaussian) for the data shown in A. Blue and red symbols indicate the response versus random error for individual trials. Grey dots indicate the proportion of “right” responses for binned trials (bin size  = 10mm). The size of the dots is indicative of the number of trials taken into account for each bin. The solid curve indicates the obtained psychometric fit from which we obtained the perceptual noise 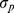. C) the perceptual noise 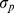 for the left/right discrimination of the errors (red data point corresponds to the example 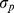 from B). For comparison, the dashed line shows the average across participants for 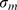, i.e. the standard deviation in random pointing error. Bars and error bars indicate the median and 25% and 75% percentiles across participants respectively. Individual participant results are indicated by the separate points. Closed symbols indicate participants with significant knowledge of their random error; open symbols participants whose performance did not differ from chance. D) The standard deviation in random error 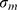. Bars and error bars indicate the median and 25% and 75% percentiles across participants respectively. Individual participant results are indicated by the separate points.

The random error for each movement was obtained by subtracting an estimate of the systematic pointing error across movements (obtained through linear regression, see Figure 2A). Using the left-right responses as a function of the random pointing error, we then computed Just Noticeable Differences (JND) to determine participants' knowledge about their random errors (Figure 2B). The JND directly corresponds to the level of perceptual noise 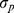 in the left-right error discrimination task. This means that 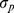 incorporates noise in the sensory feedback (e.g. proprioceptive) as well as, for instance, uncertainty in target location. Low 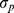 indicates good discrimination performance and high 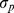 means they were unable to tell apart the different random errors.

Surprisingly, the results show that even without visual feedback participants had a good idea about their random errors when they were instructed to move as fast as possible (Figure 2C, left-most bar). In this experiment, participants were encouraged to move as fast as possible by imposing a penalty of −100 points if movement time exceeded 425 ms, which on average occurred on 12% of all trials.

Where does this knowledge come from? It could be that by forcing participants to point as quickly as possible, there was no time for correcting the error during the movement despite the knowledge about it.

To this end, we next relaxed the time constraint and instructed participants to go as slowly as necessary while being as precise and accurate as possible. To force participants to move slowly a penalty of −100 points was given if the movement duration was shorter than 1 sec. We expected this would decrease their random pointing errors (speed-accuracy trade-off) such that judgements about the remaining error should become impossible. Contrary to our prediction, we found that the random pointing errors did not decrease and that participants were still able to tell about their errors, as indicated by the low 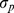 in Figure 2C. This indicates that even when moving slowly, participants did not correct online despite knowledge of the error at movement end-point.

To investigate how robust to target uncertainty the knowledge about the random errors is, we manipulated the mapping between target location and pointing response. Participants performed the same tasks with the difference that now they were not pointing *directly* at the target on the screen, but instead responses were now made *indirectly* on a graphics tablet, which was mounted flat on the tabletop (Figure 1B). This implies dissociation between target location and pointing response, which requires an additional transformation in the action planning. The reasoning behind this manipulation was that it is likely to introduce additional noise and uncertainty in the pointing movements. Surprisingly though, random pointing errors were not significantly different (F(1,11) = 0.012, p = 0.91) between the indirect and direct pointing conditions (Figure 2D). However, when participants performed the left-right judgement task in the indirect condition they performed significantly worse (Friedman 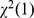  = 5.0, p = 0.025, Figure 2C), but still on average above chance (i.e. no complete loss of knowledge). This indicates that the additional transformation influences the knowledge of random errors without actually changing the pointing performance itself.

**Figure 3:**
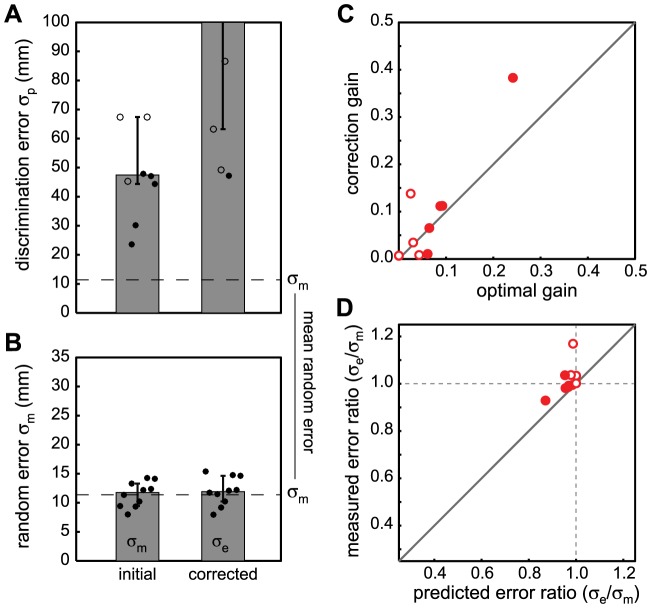
Results Experiment 2: secondary corrections. A) and B) shows the perceptual noise 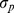 for the left/right discrimination of the errors before and after making secondary corrective movements and the standard deviation in random error 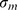 respectively. The median and 25% and 75% percentiles across participants for each condition are indicated by the bars and the error bars respectively (median and 75% percentile for the 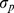 after correction are 144 mm and approaching infinity respectively). The separate points indicate the results for individual participants (filled symbols indicate performance significantly above chance). C) The measured correction gain vs. the optimal gain for each participant. The predicted optimal gains are generally relatively low which is consistent with participants' behavior. D) Measured ratio between the standard deviations in final endpoint and initial error versus the predicted error ratio. Values below one mean that participants' variance was reduced after making the corrections. Values of one mean no change and values above one mean participants' performance became worse by making corrections. As predicted from the level of perceptual noise and the resulting low correction gains participants hardly improve through correcting.

In neither *direct* nor *indirect* pointing conditions do we see an improvement in pointing performance when moving very slowly, despite the apparent knowledge of the random errors. Can it be that the random errors can only be reliably assessed at the end of the movement? If so, then forcing participants to make corrective movements after the pointing movement is complete, should lead to a decrease in random pointing error (after correction) and loss of knowledge about the random error, i.e. an increase of 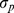 after the correction. Thus, in Experiment 2 participants were allowed to make corrective movements towards the target, after completion of the first pointing response. This experiment was performed only with the direct mapping using the touch screen. In this case (see Figure 3A), participants performed significantly worse (virtually at chance) in the left-right discrimination task (higher 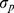) after having made corrective movements (Friedman 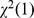  = 6.4, p = 0.011). Surprisingly though, pointing precision in terms of random error itself did not change significantly (paired t-test t(9) = −0.79, p = 0.45), i.e., participants did not become better by correcting for the perceived errors (Figure 3B). As it turns out, the corrections being made, when present, were relatively small with respect to the actual pointing error. The correction gain (correction/actual error) that participants applied varied between 38% and only 1% (see Figure 3C). These low corrections gains explain why the pointing performance after the corrective movement did not become much better. However, it leads to the next question of why the gains were so low.

**Figure 4:**
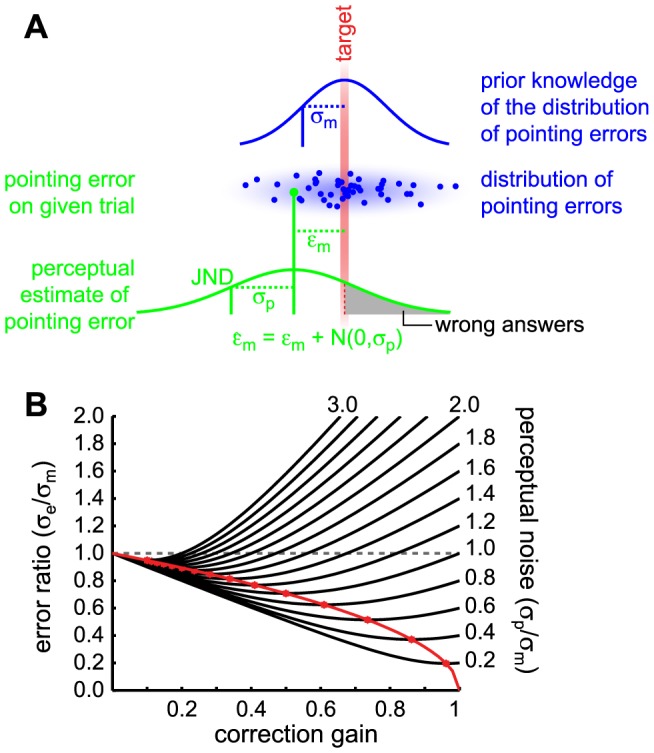
Optimal gain model. A) Error estimation model. The random errors are Gaussian distributed. For any given error 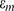 as indicated by the green dot the estimate for that error 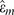 is contaminated by perceptual noise of the form 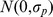. Depending on the current perceptual noise instance the participant will either answer correctly for the left/right judgement task (white area under the curve) or incorrectly (grey area). The higher the variance in perceptual noise compared to the motor noise the higher the chance of perceiving the error incorrectly in which case correction movements would lead to bigger errors on average. Thus, to be able to correct for the perceived error in an optimal way to minimize end-point variance the level of perceptual noise 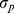 has to be weighed against prior knowledge of the distribution of the pointing errors 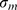. B) Theoretical ratio between the standard deviations in final endpoint after correcting and initial error versus the correction gain for several different levels of perceptual noise. Values below one mean better performance after making the corrections. Values above one mean worse performance. Optimal gains can be estimated by determining the gains for which the end point variance after correcting is lowest (red curve).

In other words, if people have knowledge about their random error, as the results indicate, why do they not fully correct for it? We expected that subjects would correct for their known error in order to minimize the variance in final end-point position, for which the visuomotor system has been shown to be optimized [12]–[16]. Naively one would think that performing corrections should naturally lead to lower variances at the end-point. However, depending on the magnitude of the different noise sources involved – i.e. the random motor error and the uncertainty in the percept of this error – the situation may be different. In fact, we can easily show that the variance in the end point distribution may get worse when making “corrections”. To understand this, it is necessary to take a closer look at the different noise sources involved and thus, at how the decision for a correction is reached. On any given trial, the final end-point error after correction (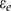) will be given by the error before correction (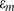) minus the estimate of this initial error (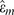) that is used for correction (see Figure 4A). However, the estimate of the error (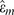) itself is contaminated by perceptual noise of the form 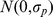, which means that, if the estimate is fully corrected for, the end points would also be fully contaminated by this perceptual noise. In other words, if the standard deviation of the perceptual noise (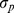) is large compared to the pointing errors themselves (i.e. the motor noise 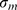), making “corrective” movement on average leads to even bigger errors in end-point positions. To prevent this, it is reasonable that the system should not fully correct for the perceived error, but apply a correction gain 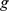 that controls how much of the perceived error to correct for. In mathematical form this can be written as:
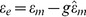
(1)

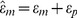
(2)


Where 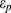 is the perceptual error from the distribution 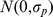. Assuming that the perceptual noise is independent of the motor noise (i.e. zero covariance), the variance across trials in the final end point (mean squared end-point error) can be written as follows:
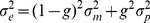
(3)


Here 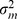 is the variance in the initial random errors across trials and 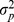 the variance of the perceptual estimates of the error as noted above. From this equation it is clear that the correction gain has a major influence on the final end-point variance. A gain of 1 means that a corrective movement covers the full extent of the perceived error, and the variance in the final endpoint is equal to 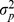. In this case, if 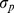 is larger than 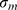, the final end-point precision is worse after making the correction. A gain of 0 means that no corrections are being made and the final end-point variance is simply 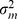. Gain values between 0 and 1 indicate that corrections are being made but the perceived error is only partially corrected for.

To illustrate more clearly how the standard deviation of the end-point distribution changes with the perceptual noise 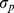 and the correction gain 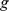, Figure 4B shows the noise ratio 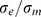 as a function of these variables. Values below 1 mean that there is an improvement in end-point variance, whereas values above 1 mean that performance gets worse when correcting. It can be seen clearly that, depending on the perceptual noise, making corrections does not always lead to better performance. The larger the perceptual noise in the error estimate, the more likely the final end-point variance instead becomes worse if the correction gain is high. Thus, depending on the perceptual noise, there is an optimal gain for which the end-point variance will be minimal as shown by the red dots and line. The optimal gain leading to a minimal end point distribution can be derived from the derivative of Equation 3. Doing so the optimal gain can be shown to scale with the motor and perceptual noise as follows:
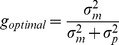
(4)


Using the optimal gain to correct for the perceived error (i.e. substituting Equation 4 into Equation 3) the minimal possible end-point variance becomes:
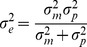
(5)


Thus, to behave optimally, people need to have knowledge about the distributions of the perceptual and motor noise, besides having an estimate of the current random error. Note that Equations 4 and 5 fit well within the Bayesian context for optimally combining noisy sources of information. Bayesian models, or derivations like Maximum Likelihood Estimation (MLE) and the Kalman filter, that operate on the same variance minimization principles, have been very successful in describing human perception of cue combination [2]–[5] as well as human sensorimotor behavior [6]–[9]. In other words, the model suggested here fits well within the Bayesian framework, which is often used to model ideal observer perception and behavior.

Since we know 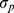 (the JND) and 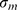 (the motor noise) from our experiment, Equation 4 provides us with a parameter-free prediction of the optimal gain for each participant, which can be compared with their actual empirical gain as in Figure 3C. The results show that, without visual feedback, the optimal gain is generally relatively low for our participants. This is because the variance in the perceptual estimate is roughly four times higher than the variance of the motor noise. Thus, the motor-result is weighed much more heavily than the perceptual estimate of the error and only tiny corrections are being made. In other words, our participants seemed to adhere to the best possible gain given their perceptual performance. Most interestingly, the fact that participants hardly corrected is simply because the variance in end-point positions would have become worse if they had corrected more.

**Figure 5:**
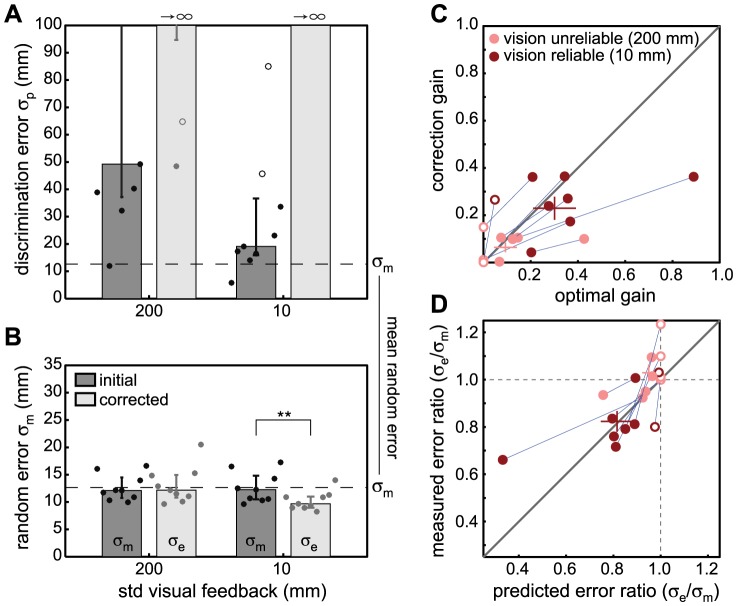
Results Experiment 3: visual feedback. Similar to Figure 3 except that participants received either unreliable or reliable visual feedback of their initial pointing position. A,B) shows the perceptual noise 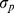 for the left/right discrimination of the errors and the motor noise 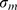 respectively, before and after making secondary corrective movements. The median and 25% and 75% percentiles across participants for each condition are indicated by the bars and the error bars respectively (medians for 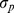 after corrections approach infinity). The separate points indicate the results for individual participants (filled symbols indicate performance significantly above chance). The dashed line shows the average across participants for the standard deviation in initial random pointing error 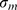. C) Behavioral gain vs. the optimal gain for each participant for the two correction conditions with visual feedback. With more reliable visual feedback correction gains generally increase as predicted. D) Behavioral endpoint versus initial error ratio vs. the predicted error ratio. With more reliable visual feedback end-point variance is reduced the most.

Thus these results are in line with keeping the final end-point variance as small as possible. With more reliable perceptual estimates of the error (i.e. smaller 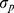), the gains should increase, and the end point variance consequently should decrease. To verify this prediction, Experiment 3 was conducted in which we gave participants different degrees of visual feedback about their pointing position prior to the corrective movement. The visual feedback was briefly flashed once upon impact when participants had completed the initial movement. The visual feedback had the form of a vertical white line with a Gaussian profile in the horizontal direction. Using blur, the horizontal Gaussian profile could be manipulated to provide either reliable (small standard deviation, 10 mm, of the Gaussian profile) or unreliable (large standard deviation, 200 mm, of the Gaussian profile) information about participants' initial pointing position, thereby manipulating 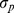. If the precision of the feedback plays a role for being able to correct for the perceived errors, participants should correct more in the reliable visual feedback condition than in the unreliable visual feedback condition. Figure 5 displays the results for this experiment. Indeed, when visual feedback is reliable, knowledge about the random error is more precise compared to when feedback is unreliable (Figure 5A), and the correction gain becomes higher (Figure 5C) in line with optimal performance. Here, now after corrections also the end point variance has significantly decreased (paired t-test t(8) = 3.95, p = 0.004, see Figure 5B,D). Thus, the more reliable the feedback about our initial pointing positions is, the more precise our knowledge about the random errors will become and the better we can correct for them.

The correspondence between the optimal gain predictions and the behavioral gains is striking, considering the measurement noise in both optimal gain predictions as well as the gain results. Furthermore, in the model we omitted additional noise sources, such as for instance the added noise that is inherent in the movement execution also of the corrective movement. Nevertheless, the identity line between experimental results and model predictions explains 80% of the complete variance across all experiments and thus we can conclude that participants' behavior is indistinguishable from optimal performance.

## Discussion

Humans generally behave very efficiently in a world full of uncertainty due to sensory and sensorimotor noise. We asked whether humans are aware of individual instances of their own varying random error resulting from the noise when pointing to targets. We initially expected the answer to be no, since otherwise this information should have been used to correct the movement online to the extent that any information about the remaining error is lost. To our surprise, we found that this prediction was wrong in every respect. First, participants in our experiments were well able to report about their random errors on individual movements without needing visual feedback. Second, even when participants were asked to make slow pointing movements, in principle allowing them to make corrections during the movement, their pointing performance did not improve and they still had knowledge about the random error at the end of the movement (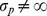). This should not have been the case if they had used that knowledge online during the movement to minimize their error. Third, when asked to make a separate corrective movement after the initial movement was complete, knowledge about the remaining error was lost even though participant's correction gains were very small. Together these findings suggest that the random error is only accessed reliably after the movement is complete (offline).

Where does the information that participants use for offline but not online corrections come from? A possible explanation for this difference is that during a movement, sensory feedback is likely less precise due to delays in the feedback-loop. Proprioception is not immediate, but is delayed by about 120 ms, which renders the sensed position of the limb inaccurate and imprecise during an ongoing movement [17]. When stationary, the sensed position from proprioception is not hampered by the delay, and becomes more precise with longer inspection times [18]. On the other hand, even delayed sensory feedback should be useful for making corrections when moving very slowly. Yet, we did not find any evidence for differences in online corrections or random error knowledge for very slow movements compared to fast movements, suggesting that the sensory feedback delay can not solely be responsible for the absence of online corrections. Another possibility is that the angle of impact with the touch screen could be responsible for providing the necessary information to discriminate individual random movement errors. Information about the impact would indeed only be available at the end of the movement. However, in a separate experiment in which participants used a laser pointer to point to targets, thus avoiding any physical contact upon movement completion, participants' knowledge of their own random errors was still well above chance, indicating that impact information does not play a key role in determining one's random errors (see File S1 and Figure S1). Rather, the difference between online and offline knowledge about our errors, may be the result of more fundamental differences of how proprioceptive feedback is used when stationary compared to during a movement. For instance, there are several reports that during hand or arm movements tactile sensitivity of the hand is reduced (sensory suppression/gating) [19]–[22]. Our results here suggest the same could be true for proprioceptive information. It has for instance been shown that proprioception at least in part relies on information from the same mechanoreceptors as touch through skin stretch [23]–[25]. Such reduced sensitivity during arm movement can explain why online knowledge of a random error is not precise enough to make corrections. Only after the movement is complete, when the hand is stationary again, information about the position of the limb becomes more precise and access to the random errors in end-point position becomes available. The gained precision in the position information when stationary, can thus be used for making discrimination judgements (better than chance) and for making corrective movements (Experiments 2 and 3).

To conclude, we have shown that, contrary to our expectations, humans do have knowledge about each individual random error, but only at the end of each movement, even when movements are very slow. It is important to note, that this knowledge about each individual error goes beyond just knowing the overall variance of the noise distribution as most models of motor control assume [6], [7], [26], [27]. In fact, the knowledge of the individual errors as shown here, may well serve as the building blocks to learn the overall noise distribution. Such knowledge about the general noise distribution becomes necessary when correcting for the currently perceived random error, since the optimal correction gain minimizing end-point variance, is a weighting factor between the variances of the perceptual noise and motor noise. Corrections made by our participants were consistent with this optimal correction gain, even in cases where it meant not correcting at all. In other words, our results show that humans combine two types of sensorimotor noise information in a Bayes-optimal fashion: direct information in form of the sensed current random error (likelihood), with indirect (prior) knowledge about the variance of the noise distributions. The fact that they only appear to do so offline after movement completion, but not while the movement is still in progress, strongly suggests that the precision of the proprioceptive sense during movements is reduced (sensory suppression).

## Materials and Methods

### Ethics statement

The experiment was approved by the ethics committee of the department of medicine of the University of Tübingen (Germany). All participants gave written informed consent.

### Apparatus

Targets were displayed on a touch screen (ELO TouchSystems 1915L) at a viewing distance of 53 cm (see Figure 1A). Participants made timed pointing responses either with their right index finger towards the touch screen or using a stylus on a graphics tablet (AIPTEK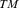 HyperPen 1200 USB). The presence of visual feedback was controlled using a pair of shutter glasses (PLATO Model P-1, Translucent Technologies Inc, Toronto, Canada). A standard USB computer mouse was modified to control the shutter glasses: when the right mouse button was held the glasses were transparent, otherwise they were opaque. Participants' head movements were restricted using a chin rest. The chin rest was aligned with the center of the touch screen or the graphics tablet, respectively.

### Stimuli, task and procedure

Visual targets were vertical red lines 1 cm wide and extending the full height of the screen. The long vertical lines reduced the pointing task to a single (horizontal) dimension. Target horizontal locations were randomly chosen from trial to trial from a range between 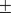
10 cm relative to screen center. Participants initiated target onset by pressing the right mouse button with their right hand (see Figure 1C). The button press starting the trial also caused the shutter glasses to become transparent so that participants could see the target. By continuously holding down the mouse button participants could inspect the target as long as they wanted before the onset of the pointing movement. When they started the movement, releasing the mouse button, the shutter glasses became opaque, preventing visual feedback. When the pointing movement was complete, upon touching the touch screen (or tablet), participants were notified by a beep that they could then respond by button press, using their left hand, whether they thought they had landed left or right of target. After the response had been made, a second beep notified participants to press the right mouse button again with their right hand. Thereby the glasses became transparent again and participants could see the score they received written in big letters on the screen. The score was based on their absolute accuracy, thus not providing error feedback with regard to the direction of the error. The next target then appeared automatically after 1 sec. The score after each trial was used to motivate participants to point as accurately as possible despite having to do the additional left-right judgement task. The participant scored 100 points if the absolute pointing error was below 1 cm; 50 points when between 1 and 2 cm; 25 between 2 and 3 cm and 0 otherwise. Penalties of −100 points could be incurred if a movement was not completed within the time limit set for the condition (fast or slow – see below). To further motivate participants to point as accurately as possible end-scores for each block of trials were entered into a high-score list shown at the end of each block using an alias for each participant to ensure anonymity.

### Experiment 1: Knowing the error

In Experiment 1 two factors were varied. One factor was the complexity of the mapping between the visual target and the required motor response. In the “direct” condition participants made directed movements towards the target displayed on a touch screen. In this case the mapping between target and required movement is natural. In the “indirect” condition targets were displayed on the touch screen as before, but participants were instructed to make the pointing responses on a graphics tablet using a stylus (see Figure 1B). This meant that the mapping between the visual target location and the corresponding movement is indirect and more complex. That is, participants had to mentally project the left-right location of the vertical display screen into a left-right response on the horizontal tablet (Figure 1B). We expected this to insert more uncertainty in required movements and therefore that participants should perform worse in terms of both the pointing precision and the left-right judgement task. The mapping except for the 90 deg rotation was 1-to-1, such that the scale of the pointing area was the same and aligned with the visual area. In the “indirect” condition the participants held the stylus in their hand continuously, but they used a preferred finger of the same hand to press the mouse button to start each trial.

The second factor that we varied was the speed of the pointing movement, i.e. the time between the release of the mouse button and touching the touch screen or tablet. In the “fast” condition the participants were instructed to make fast movements below 425 ms. If they were too slow they would receive a penalty of −100 points (negative overall scores were possible). In the “slow” condition they were instructed to make slow movements of above 1.0 sec and a penalty of −100 points was incurred if they moved too fast. The slow condition allowed participants to correct online for errors they might make along the way.

This 2–by–2 design resulted in four conditions. In each of the four conditions the participants performed 100 trials. Between conditions the participants were required to take a short break. The order of the conditions was counterbalanced across participants.

### Experiment 2: Correcting for the error

In Experiment 2 we investigated the influence of corrective pointing movements which participants were instructed to perform after the initial movement was completed. After the corrective movement participants indicated by button press (with their left hand) when they were satisfied with their current position. There was no time limit in this condition. Further procedures for the left/right discrimination task and scoring were the same as for Experiment 1.

### Experiment 3: Manipulating the correction gain (pointing with visual feedback)

We investigated if correction gains improved with visual feedback of the pointing endpoint. For this experiment, to be able to render visual feedback while varying its reliability, we used a different setup: a large back projection screen (220 by 176 cm) in an otherwise dark room. Participants were seated behind a custom-made rack. On the first level of the rack a graphics tablet (WACOM Intuos 3 A3-wide; active area 48.8 by 30.5 cm and a grip pen) was placed in order to record the pointing behavior of the participants. A second level of the rack draped in black cloth prevented the participants from seeing their own arm or the graphics tablet while performing the pointing task. The head movements of the participants were restricted by a chin rest. The viewing distance to the screen was 53.5 cm. A horizontally centered semi-circular area (radius of 25 mm) on the lower edge of the tablets active area provided a trigger to start trials, triggering target onset, instead of the mouse button used in Experiments 1 and 2. This area was also indicated by a physical ring attached to the tablet to help locate this area without seeing the hand. The target was extinguished as soon as the participant lifted the grip pen from the tablet or moved outside this semicircular area, indicating movement onset.

The task was the same as in the correction condition of Experiment 2. The only major difference is that at the moment of first touching the graphics tablet visual feedback of their current position was briefly provided (100 ms) after which participants could make the corrective movement. The visual feedback was in form of a vertical long white line (Michelson contrast 0.26) with a Gaussian profile in the horizontal direction. Standard deviations of the profile were either 10 mm (reliable visual feedback) or 200 mm (unreliable visual feedback). Trials from these two conditions were presented intermixed in random order in 2 separate blocks of 100 trials.

### Participants and training procedure

There were 12 (mean age of 26.5 years), 10 (mean age 25.5 years) and 9 participants (mean age 27.9) for Experiments 1, 2 and 3, respectively. All participants were right-handed. Before the experiments started, participants were familiarized with the task and pointing movement mappings in short training blocks of 50 trials. In these training blocks the procedure was the same as during an experimental block except that participants were not required to do the left-right judgement after each movement. Also participants received feedback of where they had landed after each training trial. The feedback consisted of a high contrast vertical line with a Gaussian intensity profile in the horizontal direction (standard deviation of the profile was 5 mm).

### Analysis

As noted in the introduction pointing errors consist of two parts: systematic errors due to miscalibrations in the visuomotor system and random errors due to noise that causes the movements to vary even if the planning of the movement is the same. Here we were interested in the second type of error, the random error, only. Thus, in order to investigate whether participants knew about their random errors we needed to separate the pointing errors into its two parts: the systematic and the random errors. Systematic errors in the pointing movements can be determined by looking at the averages across trials and were found to be linearly dependent on target location. This dependency was identified by linearly regressing pointing errors with respect to target locations. We verified that the regression slopes did not vary over time, indicating that participants had no knowledge of their systematic errors (Figure S2). The residuals of the linear fit to the data as a function of target location were taken as the random error (Figure 2A).

To investigate whether participants had knowledge about this random error, we looked at the left/right-response as a function of the size of the random errors. If participants have knowledge about the random errors they will have answered “left” more often when the random error was to the left and “right” when the random error was to the right. The bigger the error the higher the probability they give the “correct” response. Instead if they do not have access to the random error, the participants should be performing at chance in this left/right judgement task (i.e. 50% “right” responses) regardless of the magnitude of the pointing error. To quantify the precision with which participants can tell about the random pointing error, we fitted cumulative Gaussians to participants' responses using the Psignifit toolbox for Matlab [28] and determined the Just Noticeable Difference (JND – Figure 2B). The JND directly corresponds to the standard deviation 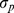 of the underlying Gaussian distribution. Sometimes, large negative JNDs occurred when performance was close to chance. These were set to positive infinity to ensure singular values for the JNDs at chance performance.

To verify whether an individual 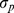 indicates performance significantly different from chance, each 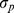 was compared to the distribution of possible outcomes for 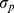 under the Null-hypothesis of chance performance. The distribution under the Null-hypothesis was obtained through Monte Carlo simulations. If 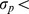 4.36 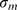 the chance of the individual 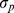 belonging to the distribution under the Null-hypothesis is less than 5% indicating significant knowledge of the random errors. Statistical analysis for comparing 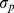
's across conditions was done using the Friedman test (for non-parametric repeated measures).

## Supporting Information

Figure S1
**Results control experiment: pointing without impact.** A) Shows the left/right discrimination 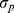 for the control experiment. The separate points indicate the results for individual participants. Closed symbols indicate participants with significant knowledge of their random error; open symbols participants whose performance did not differ from chance. The median and 25% and 75% percentiles across participants for the 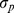 are indicated by the bar and the error bars respectively. The dashed line shows the average across participants for the standard deviation in random pointing error. B) The standard deviation in random error. Individual participants results are indicated by the separate points. Dashed line and error bars indicate the median and 25% and 75% percentiles across participants.(EPS)

Figure S2
**Absolute regression slopes for the systematic errors over consecutive blocks of 20 trials** (fast and direct pointing condition of Experiment 1). If participants knew their systematic errors they should correct for them over time to increase their accuracy. This would mean that the systematic error slope should decrease over time. However, there was no difference between the separate blocks of trials (Friedman 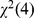  = 2.47; p = 0.65). This means that participants did not correct for their systematic errors, indicating no knowledge of systematic errors.(EPS)

File S1 **Control experiment: Pointing without impact.**
(PDF)
