# Supplementary material for: Knowing Each Random Error of Our Ways, but Hardly Correcting for It: An Instance of Optimal Performance
Source: PLoS One. 2013 Oct 30;8(10):e78757. doi: 10.1371/journal.pone.0078757 (PMC3813602; doi:10.1371/journal.pone.0078757)
Supplement: File S1 — Control experiment: Pointing without impact. (PDF) [file pone.0078757.s003.pdf]

Supporting information to: Knowing each random error of our ways, but hardly correcting for it: An instance of optimal performance. Loes C.J. van Dam, Marc O. Ernst

Pointing without impact

Introduction

The results of the main experiment indicate that participants have knowledge about their random error when performing pointing movements. As pointed out in the main text it seems that the random error is assessed only after the movement is complete and not during the movement. This could be indicative that the impact of the finger with the touch screen (for instance through touch angle), provides the crucial feedback to identify the error. We performed a simple control experiment in order to verify whether the impact of the finger with the touch screen is indeed providing the crucial feedback or not.

In order to do so, we had participants perform the same pointing and left-right judgement tasks, but this time they pointed using a laser pointer to targets presented on a sheet of paper. In this case there is no impact with the plane in which the targets are being presented at the end of movement. Thus if the impact is providing the crucial feedback to identify the error in the main experiment, participants should not be able to identify their random errors in this control experiment.

Setup and Procedure

5 targets (red vertical lines of length 59.5 cm and width 4 mm) were spaced 12 cm apart on a big sheet of paper (118.5 by 81 cm) that was pinned to a poster board. The 5 targets were numbered 1 to 5 from left to right and at the beginning of each trial the experimenter would call out one of the numbers to indicate the next target to point to. The participant stood approximately 1.2 m from the poster board with a laser-pointer in the preferred hand and wearing shutter glasses or darkened goggles that could be manually pulled up or down to control for visual feedback. The glasses and goggles were prepared such that they covered the whole field of view. When the target number was called the participant could view

the target and, after the participant indicated verbally that he/she was ready to make the movement, further visual feedback was prevented by pulling down the goggles or making the shutter glasses become opaque. The participant was instructed to make a fast pointing movement without trying to correct for it afterwards, although slight movements of the limb after the initial movement could not completely be prevented. After the movement was complete the experimenter measured the pointing error by reading of the position of the laser point relative to the target (thin gray lines spaced 1 cm apart on the target sheet aided the experimenter in doing so). Also as before in the main experiment the participants were asked if they thought they had landed left or right of the target and the experimenter wrote down their answer. After the participants had lowered their arm they were allowed to pull up the goggles to prepare for the next trial.

As before in the main experiment participants performed 100 trials in a single block. Target order across trials was randomized but immediate repeats of the same target were prevented. In order to motivate participants to point as accurately as possible, and thus to prevent them from making deliberate errors to render the left-right judgement task easier, subjects were told that the participant with the best pointing performance would win a prize.

Participants

The experiment was conducted during an annual retreat of the Human Perception, Cognition and Action department of the MPI for Biological Cybernetics. Ten participants took part in this experiment, all of whom were employed by the MPI but naive as to the purpose of the experiment. Before the actual experiment started the participants were allowed 10 practice trials in which they also received visual feedback at the end of the movement (i.e. the goggles were pulled up before the participant lowered their arm after the pointing movement). Note though that the practice period was not as extensive as in the main experiment due to a limited amount of time available per participant.

Analysis

The analysis that was performed on the data in this control experiment was very similar to the analysis of the main experiment with two exceptions. First pointing errors were converted to error in deg rather than mm. This corrects for the fact that angular errors in the pointing movement lead to larger errors on the target sheet for more extreme targets relative to central targets, due to the more skewed projection of the laser pointer onto the target plane.

Second, before identifying systematic errors and random errors we performed an analysis of the errors over time. It was found that, contrary to the results of the main experiment, in this experiment the pointing errors drifted over time at least for some participants. This was likely due to the fact that head and stance position etc were not fixed during this experiment such that participants could change their stance and limb angles during the ongoing experiment. To correct for these effects the raw pointing data was low pass filtered (4th order Butterworth with a cutoff frequency of 0.02/trial) to obtain the drift over time. This drift was then subtracted from the raw data to remove these temporal effects. After this initial step the further data analysis was exactly the same as for the main experiment.

Results and Discussion

The results of this control experiment are portrayed in Figure S1. Figure S1A shows σ_p for each participant (single points) as well as the median and 25% and 75% percentiles in σ_p across participants. For comparison to the pointing precision the standard deviations for each participant and median across participants are shown in Figure S1B. Note that the experimental settings were not as well controlled as in the main experiment described in the main text. For instance subjects' stance and pointing limb angles were not fixed and end point positions were not always stable while participants tried to assess their random error. Also measuring the pointing errors was not automated as in the main experiment but read off by the experimenter which is more likely to introduce mistakes.

Nevertheless the results show that at least there were some subjects who performed reasonably well in the left-right judgement task without having obtaining additional feedback of an end-point impact.

Thus this experimental outcome can be considered as a proof of principle that the end-point impact is not crucial to identifying ones random errors.
